# Knowledge level and constructs of the theory of planned behaviour (TPB) to the practice of unsafe abortion among postnatal mothers attending Mkonze health center, Dodoma Region, Tanzania

**DOI:** 10.1186/s12889-024-18921-z

**Published:** 2024-05-28

**Authors:** Immaculata Alphonce Samila, Joanes Faustine Mboineki

**Affiliations:** Department of Nursing Management and Education, School of Nursing and Public Health, The University of Dodoma, P. O. Box 259, Dodoma, Tanzania

**Keywords:** Abortion, Unsafe abortion, Knowledge, Attitude, Perceived behavioral control, Subjective norms, Sociodemographic characteristics, Practice

## Abstract

**Background:**

Unsafe abortion is now a global agenda because 45% of all global abortions are unsafe, and 97% are occurring in developing countries. In Tanzania, one million reproductive-aged women face unplanned pregnancies per year, and 39% end up with abortion. About 16% of maternal deaths are reported per year in Tanzania, and unsafe abortion takes the second position. There are several efforts to prevent and intervene unsafe abortions, such as equipping healthcare facilities across all levels of healthcare, approval of Misoprostol use, establishment of comprehensive post-abortion care (PAC), revising policy guidelines and standards, provision of emergency contraceptives, and capacity building of healthcare providers. There is little documentation about how the constructs of the theory of planned behaviour, knowledge, and sociodemographics influence the practice of abortion.

**Objectives:**

To assess the association of knowledge level, sociodemographic characteristics, and constructs of the theory of planned behaviour (TPB) to the practice of unsafe abortion among postnatal mothers at Mkonze Health Center in the Dodoma region.

**Methodology:**

It is an analytical cross-sectional study design conducted in Dodoma-Tanzania and involved 206 postnatal women. A validated questionnaire was used and analysis was performed in the Statistical Package for the Social Sciences (SPSS), through descriptive and inferential statistics.

**Results:**

The practice of unsafe abortion in the current study is 28/206 (13.6%), influenced bytheir lower educational level and being single women. It was found that the majority had adequate knowledge of unsafe abortion 129 (62.6%), positive attitude 130 (63.1%), good subjective norms 113 (54.9%), and positive perceived behavioral control111 (53.9%). Knowledge, attitude, subjective norms, and perceived behavioral control were not significantly associated with the practice of unsafe abortion.

**Conclusion:**

The majority of the respondents had high knowledge, attitudes, subjective norms, and perceived behavior control on unsafe abortion. This is an indicator that the implemented initiatives are effective. Maintaining the ongoing effort and improving strategies are promising to mitigate the burden of unsafe abortion. Future research needs to find out hidden factors associated with attitude and how health beliefs might influence someone’s attitude towards unsafe abortion.

**Supplementary Information:**

The online version contains supplementary material available at 10.1186/s12889-024-18921-z.

## Introduction

Abortion is a global issue that has been taking the attention of different cultures, religions, and social, psychological, and country laws [1]. Abortion has become a major issue across the country’s boundaries whereby some countries’ policies accept it in special scenarios such as life-threatening conditions and emergencies while others such as United States and Canada allow women to abort depending on their reasons such as medical, psychology, rape, financial hardship, incest, and fetal abnormalities [2]. Out of 210 million pregnancies per year, 46 million pregnancies end in abortion [3].

Unsafe abortion is now a global agenda because 45% of all global abortions are unsafe, with 97% occurring in developing countries and 3% in developed countries [4]. Out of 55.7 million abortions that occurred globally between 2010 and 2014, 25.1 million of these were unsafe every year [5]. It is approximated that there are 20 million unsafe abortions annually and one out of 250 end to death and maternal disabilities [6]. This is supported by another study reporting that 13% of unsafe abortions end in maternal death each year [7]. While a huge burden of unsafe abortion is in Sub-Saharan countries, East Africa accounts for 75% of unsafe abortions [8].

Different reasons contribute to the decision of unsafe abortion among many women and adolescent girls, including unpreparedness for pregnancy, poor access to contraceptives, education level, and sexual education, economic issue, rape, marital status, victim of incest, support from partner and parents, society norms, religion, stigma, health care system and laws of the countries [9]. All these have contributed to unsafe abortion among women in countries where there is no accessibility of safe abortion due to restrictive laws of abortion [10]. The rate of unsafe abortion is increasing proportionally especially in restrictive countries due to the influence of demographics, low community awareness, socioeconomic and cultural factors such as financial issues, and peer groups, to continue their education, secure their future aspiration, relationship status, stigma, risk measuring, and availability and accessibility of methods of abortion such as misoprostol [11]. According to research done in Zambia, 77 women out of 362 had unsafe abortions whereby most of them fall into the group of unmarried, living in high density, having a history of unplanned pregnancy and a history of miscarriage [12]. Generally, the higher proportions of unsafe abortion are confined to countries with highly restrictive abortion laws [13, 14], and countries with difficulty accessing health facilities [15]. Abortion is the termination or expulsion of the fetus before 20 weeks of gestation [16]. Abortion can be spontaneous or induced, whereby it is induced abortion when there is termination of unwanted pregnancy by use of external methods (medically or by dilation and curettage) [17]. On the other side, spontaneous abortion refers to a miscarriage or purely accidental abortion [18]. When the abortion is carried out using a method recommended by WHO, appropriate to the pregnancy duration, and by someone with the necessary skills, it is considered a safe abortion [19]. Meanwhile, unsafe abortion refers to a procedure of pregnancy termination either by persons lacking the necessary skills or in an environment that does not conform to minimal medical standards or both [7].

In Tanzania, one million reproductive-aged women face unplanned pregnancies per year, and 39% end up with abortion [20]. About 16% of maternal deaths are reported per year in Tanzania, and unsafe abortion takes the second position [21]. Several efforts are observed in Tanzania to combat unsafe abortion by improving post-abortion care and provision of emergency contraceptives and safe abortion services to women underwent incest and rape [8]. In addition, the efforts of tackling unsafe abortion in the country have been through identification and prioritizing unsafe abortion services to zones with the highest rates of abortion, promotion of men’s involvement in family planning, equipping healthcare facilities across all levels of the healthcare system to provide basic post-abortion care, implementation of national Road Map Strategic Plan, approval of Misoprostol, and building on a comprehensive post-abortion care (PAC) training program [22]. Moreover, there have been new and revised policy guidelines and standards to guide the extension of post-abortion care, capacity building of healthcare providers, developing networks, and platforms to promote understanding of abortion issues [23]. It is reported that one-third of hospital admissions due to pregnancy complications are due to unsafe abortion while one-quarter lead to maternal death [20]. Unsafe abortion in Tanzania is due to unintended pregnancy and inaccessibility of contraceptives [24]. Most studies in Tanzania regarding abortion have focused on incidence, abortion-seeking practices, and service delivery [21]. There is little documentation about how the constructs of the theory of planned behavior and knowledge influence abortion practice. Therefore, the study aimed to assess the association of knowledge level, sociodemographic characteristics, and constructs of the theory of planned behaviour (TPB) to the practice of unsafe abortion among postnatal mothers at the Mkonze health center in Dodoma region.

The theory of planned behavior (TPB) which is a cognitive theory by Azjen (1985), states that an individual’s decision to engage in a specific behavior can be predicated by their intention to engage in that behaviour [25]. The theory has four constructs; attitude, subjective norms, perceived behavior control, and intention [26]. The specific objectives of this study are; ① To determine the knowledge about unsafe abortion among postnatal mothers at Mkonze health center in Dodoma regional ② To assess the attitude towards unsafe abortion among postnatal mothers at Mkonze health center in Dodoma regional ③ To predict subjective norms leading to unsafe abortion among postnatal mothers at Mkonze health center in Dodoma regional ④ To determine perceived behavior control about unsafe abortion among postnatal mother at Mkonze health center in Dodoma regional and ⑤ To evaluate the intention practice towards unsafe abortion among postnatal mother at Mkonze health center in Dodoma regional.

## Methods

### Study design

An analytical cross-sectional study design was employed to use the construsts of the theory of planned behavior and knowledge to assess the intentional practice of unsafe abortion among postnatal mothers at the Mkonze health center in Dodoma region.

### Study area

The study was carried out in Dodoma City at Mkonze Health Center. Dodoma city was chosen as the study area because it has the highest magnitude of teenage pregnancies (29–39%) compared to other regions of Tanzania [27, 28]. Moreover, it is a marked region with relatively low utilization of contraceptives [29].

### Study population

The study population included all postnatal women attending or admitted to Mkonze Health Centre. Participants were excluded if had sickness to impair their responses, could not comprehend the instructions, and were not willing to participate because of self-reported tiredness and busy.

### Sample size calculation and sampling technique

#### Sample size calculation

The sample size was calculated using the sample size formula:

n = (Z² * P * (1 - P)) / E².

Where:

n = Sample size,

Z = Standard normal deviation (for a 95% confidence interval, Z = 1.96),

P = Proportion of target population. The prevalence of 16% for unsafe abortion in Tanzania [22].

E = Marginal error on the quantity to be estimated (5%).

Therefore, the calculated sample size was 206.

### Sampling technique

Probability sampling especially systematic random sampling was used in the study. The postnatal ward at the health center had a daily recorded number of admitted postnatal women in the register. The required number of participants per day was 6 postnatal women, therefore, the nth number was calculated by dividing the number of required postnatal women over the total admitted number of postnatal women of a particular day. The participants were then selected at the interval of the nth number in the register.

### Data collection procedure and data collection tools

Data were collected from Mkonze Health Centre from 25th May to 30th June 2023 by a principal investigator with an assistant. The questionnaire which was used in the data collection was developed and validated by a principal investigator. The development started with a literature review to identify items for each variable, followed by content validity where the experts were invited to rate how well was the tool. The tool was pre-tested to 20% of the actual sample size, and internal consistent reliability was determined. Through principal component analysis (PCA), the weak items were modified, making the final tool to have consisted six parts, (1) socio-demographic information, (2) attitude (3) practice (4) knowledge (5) perceived behavior control, and (6) subjective norms among postnatal mothers. Different parts contained varying numbers of items related to the study’s context. For instance, the practice toward unsafe abortion among postnatal mothers was assessed using 8 questions, with response 1 for Yes and 2 for No, the knowledge toward unsafe abortion among postnatal mothers was assessed using 13 questions having responses of 1-Yes and 2-No.Attitude toward unsafe abortion among postnatal mothers was assessed using five items on a 5-Likert scale (1-strongly disagree,2-disagree, 3-neutral, 4-agree, 5-strongly agree). The higher the score (strongly agree) the more positive attitude of the participants. Subjective norms toward unsafe abortion among postnatal mothers were assessed using five items having a response of 1 for Yes and 2 for No. Participants who responded as “Yes” in each item, were considered to get the correct answer. Perceived behavior control toward unsafe abortion among postnatal mothers was assessed using three items 5-Likert scale (1-strongly disagree, 2-disagree, 3-neutral, 4-agree, and 5-strongly agree). The increased number indicates a correct answer, with the maximum (strongly agree).

The questionnaire was translated from English to Swahili’s native language for the convenience of participants.

### Data analysis

Regarding measures and data processing, knowledge, subjective norms, and practices of unsafe abortion were measured through binary responses of Yes/No, but attitude and perceived behavioral control were measured through a 5-Likert scale. In each variable, the average of items were computed and the scores were categorized. The cut-off point for each variable was 50%, the above 50%, the higher score, and vice versa.Data were entered and analyzed in the Statistical Package for the Social Sciences. Descriptive statistics summarize the data in the form of frequency, percentage, and mean. A cross-tabulation was carried out to look association between sociodemographic data and factors. A *P*-value of less than 0.05 was considered statistically significant.

## Results

### Social demographic characteristics of the respondents

The study recruited 206 postnatal women in the study. The majority of the respondents 56 (27.2%) were aged 25–29 followed by 52 (25.2%) aged 30–34. It was found that 159 (77.2%) of participants lived in urban areas. The participants were married 133 (64.6%), single 33 (16.0%), divorced 28 (13.6%), and windowed 12 (5.8%). Regarding the occupation, participants were entrepreneurs 82 (39.8%), peasant 67 (32.5%), employed 32 (15.5%), and housewife 25 (12.1%). Regarding the education status of the respondents, they had a primary level of education of 92 (44.7%), secondary level education of 54 (26.2%) college or university 23 (11.2%), and never attended school 37 (18%). Refer to Table 1.

**Table 1:** Sociodemographic characteristics of respondents

Variables	Frequency(n)	Percentage (%)
**Age group**		
5–19	17	8.3
20–24	34	16.5
25–29	56	27.2
30–34	52	25.2
35–39	30	14.6
40–44	17	8.3
**Place of residence**		
Rural	47	22.8
Urban	159	77.2
**Marital status**		
Single	33	16
Married	133	64.6
Divorced	28	13.6
Widow	12	5.8
**Occupation**		
Peasant	67	32.5
Employed	32	15.5
House wife	25	12.1
Entrepreneur	82	39.8
**Education level**		
Not attended formal education	37	18
Primary education	92	44.7
Secondary education	54	26.2

### The practice of unsafe abortion among the participants

The majority of the participants about two-thirds seemed not to have had an abortion in the course of their lives 161 (78.2%) and those who had abortions were about one-third 45 (21.8%) of the total participants. Of those who had abortions, 17 (38%) had safe abortions and 28 (62.2%) had an unsafe abortion. Therefore, the practice of unsafe abortion in the current study is 28/206 (13.6%). The highly useful method for unsafe was medication 89 (43.2%) and home remedies 84 (40.8%). Three-quarters did not practice unsafe abortion in the past two years 177 (85.9%) and those who practiced it were 29 (14.1%). Reasons for unsafe abortion are highly due to the advice from friends and parents 129 (62.6%), rape 97 (47.1%) pregnancies rejected by their partners 89 (43.1%), and those in need to continue with studies 88 (42.7%). From those who practiced unsafe abortion, the high frequency of having an unsafe abortion was once in a life lifetime 26 (12.6%). Refer to Table 2. A total score was computed whereas a score above 4 was regarded as good practice toward unsafe abortion and a score below 4 was regarded as poor practice toward unsafe abortion. By summing up the scores of practices, it was found that the majority of participants had practiced unsafe abortion 147 (71.4%) and 59 (28.6%) had practiced safe abortion.

**Table 2:** Practices of abortion among study participants

Variable	Frequency (*n*)	Percent (%)
Ever had abortion?		
Yes	45	21.8
No	161	78.2
It safe abortion?		
Yes	18	8.7
No	188	91.3
It an unsafe abortion?		
Yes	28	13.6
No	178	86.4
Ever used home remedies for abortion		
Yes	84	40.8
No	122	59.2
Ever used medication are used for abortion		
Yes	89	43.2
No	117	56.8
Have you had an unsafe abortion in the past two years		
Yes	29	14.1
No	177	85.9
Ever advised by friends for unsafe abortion		
Yes	129	62.6
No	77	37.4
Pregnancy resulting from rape was a reason for unsafe abortion		
Yes	97	47.1
No	109	52.9
Wanted to continue studying the reason for your unsafe abortion		
Yes	88	42.7
No	118	57.3
Pregnancy refused by Patterner as a reason for unsafe abortion		
Yes	89	43.2
No	117	56.8
Have you ever been influenced to perform an unsafe abortion		
Yes	75	36.4
No	131	63.6
Response to unsafe abortion		
Positive	29	14.1
Negative	177	85.9
Frequency of unsafe abortion		
Once	26	12.6
Twice	8	3.9
Above 2	2	1
None	170	82.5

### Knowledge of unsafe abortion among participants

**Fig. 1:**
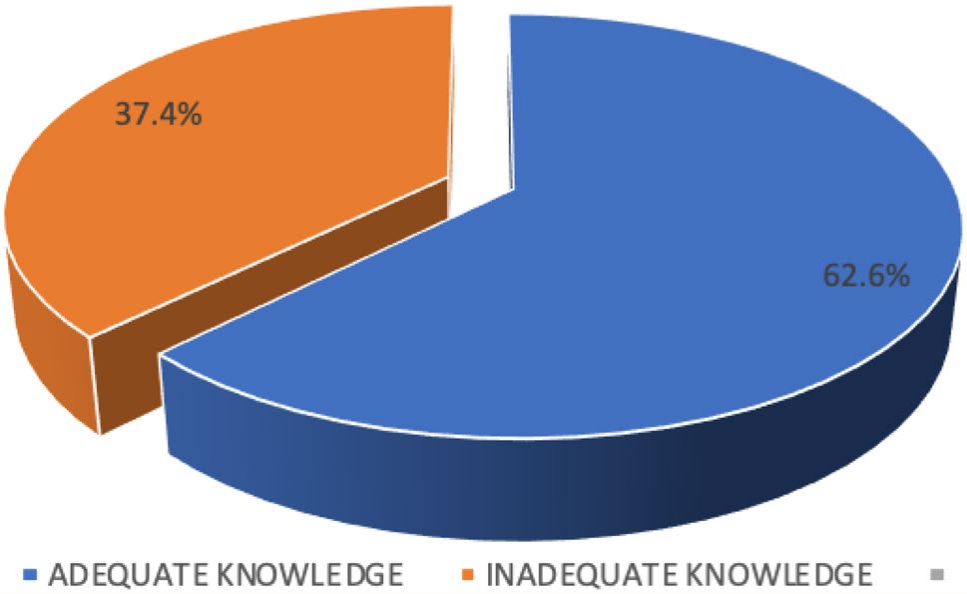
Knowledge level of unsafe abortion among study participants

Most of the participants had heard of safe 150 (72.8%), and unsafe abortion 150 (72.8%). Participants agreed that unsafe abortion is a problem in our current society 116 (56.3%). Regarding the source of information on unsafe abortion, most of the participants got information through friends 132 (64.1%), social media 116 (56.3%), health professionals 112 (54.4%), and relatives/family 114 (55.3%). It was found that 158 (76.6%) of participants had never experienced unwanted pregnancy in their lives and further responded that in case they become pregnant unwillingly they would continue and give birth 176 (85.4%). Most women knew the gestation age when abortion is unsafe to be 3 months of pregnancy 126 (61.2%). On the signs and symptoms of unsafe abortion, participants agree that the following are signs and symptoms of unsafe abortion; abdominal pain 148 (71.8%), vaginal infection 152 (73.8%), vaginal bleeding 165 (80.1%), shock 149 (72.3%), and heavy bleeding 151 (73.3%). Participants mentioned that unsafe abortion has complications 157 (66.5%) like infertility 142 (68.9%) and future pregnancy problem 133 (64.6%). The majority knew that abortion is illegal in Tanzania 151 (73.3%) and agreed with the presence of home remedies used in abortion 133 (64.6%) but reported that the use of home remedies for abortion is not safe 154 (74.8%). Refer to Table 3. A total score was computed whereas a score above 7 was regarded as adequate knowledge toward unsafe abortion and a score below 7 was regarded as inadequate knowledge toward unsafe abortion. The summation of the total score was performed to categorize variables into adequate and inadequate knowledge. Finally, the majority of the participants had adequate knowledge of unsafe abortion 129 (62.6%) and the rest had inadequate knowledge 77 (37.4%). Refer to Fig. 1.

**Table 3:** Knowledge of unsafe abortion among participants

Variables	Frequency(*n*)	Percentage (%)
Ever heard of safe abortion		
Yes	150	72.8
No	56	27.2
Ever heard of unsafe abortion		
Yes	150	72.8
No	56	27.2
Is unsafe abortion a major problem today		
Yes	116	56.3
No	78	37.9
Don’t know	12	5.8
Information obtained through social media		
Yes	116	56.3
No	90	43.7
Information obtained through health professionals		
Yes	112	54.4
No	94	45.6
Information got through relatives or family		
Yes	114	55.3
No	92	44.7
Information got through friends		
Yes	132	64.1
No	74	35.9
Ever experienced unwanted pregnancy		
Yes	48	23.3
No	158	76.7
The decision In case you have an unwanted pregnancy		
Continue and give birth	176	85.4
Terminate	30	14.6
Testation age is abortion unsafe		
After 3 months of pregnancy	126	61.2
At any time of pregnancy	80	38.4
abdominal pain is a sign and symptom of unsafe abortion		
Yes	148	71.8
No	58	28.2
Vaginal infection is a sign and symptom of unsafe abortion		
Yes	152	73.8
No	54	26.2
Vaginal bleeding is a sign and symptom of unsafe abortion		
Yes	165	80.1
No	41	19.9
Shock is a sign and symptom of unsafe abortion		
Yes	149	72.3
No	57	27.7
Heavy bleeding is a complication of unsafe abortion		
Yes	151	73.3
No	55	26.7
Infertility is a complication of unsafe abortion		
Yes	142	68.9
No	64	31.1
No complications of unsafe abortion		
Yes	69	33.5
No	157	66.5
Unsafe abortion can lead to future pregnancy problems		
Yes	133	64.6
No	54	26.2
Don’t know	19	9.2
Unsafe abortion is illegal in Tanzania		
Yes	151	77.3
No	36	17.5
Don’t know	19	9.2
There is the use of home remedies for abortion		
Yes	133	64.6
No	73	35.4
Home remedies are safe for abortion		
Yes	52	25.2
No	154	74.8

### The association between sociodemographic and knowledge level of participants

Only age was significantly associated with the knowledge of participants, such that respondents of 30–34 had adequate knowledge of safe abortion compared to other age groups (χ^2^ = 12.897; *P* = 0.024). Other sociodemographic characteristics were not significantly associated with knowledge level; place of residence (χ^2^ = 2.313; *P* = 0.128), marital status (χ^2^ = 2.248; *P* = 0.522), occupation (χ^2^ = 3.704; *P* = 0.295), and education level (χ^2^ = 0.130; *P* = 0.988). Refer to Table 4.

**Table 4:** Association between sociodemographic characteristics and knowledge level among respondents

Variables	Knowledge level Adequate *n* (%)	Inadequate *n* (%)	χ^2^ (*P*-Value)
**Age group**			
15–19	13(76.5)	4(23.5)	12.897 (0.024)
20–24	18(52.9)	16(47.1)	
25–29	18(52.9)	16(47.1)	
30–34	40(76.9)	12(23.1)	
35–39	18(60)	12(40)	
40–44	6(35.3)	11(64.7)	
**Place of residence**			
Rural	25(53.2)	22(46.8)	2.313 (0.128)
Urban	104(65.4)	55(34.6)	
**Marital status**			
Single	19(57.6)	14(42.4)	2.248 (0.522)
Married	88(66.2)	45(33.8)	
Divorced	16(57.1)	12(42.9)	
Widow	6(50)	6(50)	
**Occupation**			
Peasant	37(55.2)	30(44.8)	3.704 (0.295)
Employed	24(75) 8(25)		
House wife	16(64)	9(36)	
Entrepreneur	52(63.4)	30(36.6)	
**Education level**			
Not attended formal education	2464.9)	13(35.1)	0.130 (0.988)
Primary school	5762)	35(38)	
Secondary school	34(63)	20(37)	
College/university	14(60.9)	9(39.1)	

### The association knowledge level and practice of unsafe abortion

Among postnatal women who had adequate knowledge, 94 (72.9%) had unsafe abortion. While, those who had inadequate knowledge, 53 (68.8%) had unsafe abortions. Generally, the result has shown that there was no significant association between knowledge level and practice of unsafe abortion (χ^2^ *=* 0.385; *P* = 0.535).

### Attitudes toward unsafe abortion among participants

**Fig. 2:**
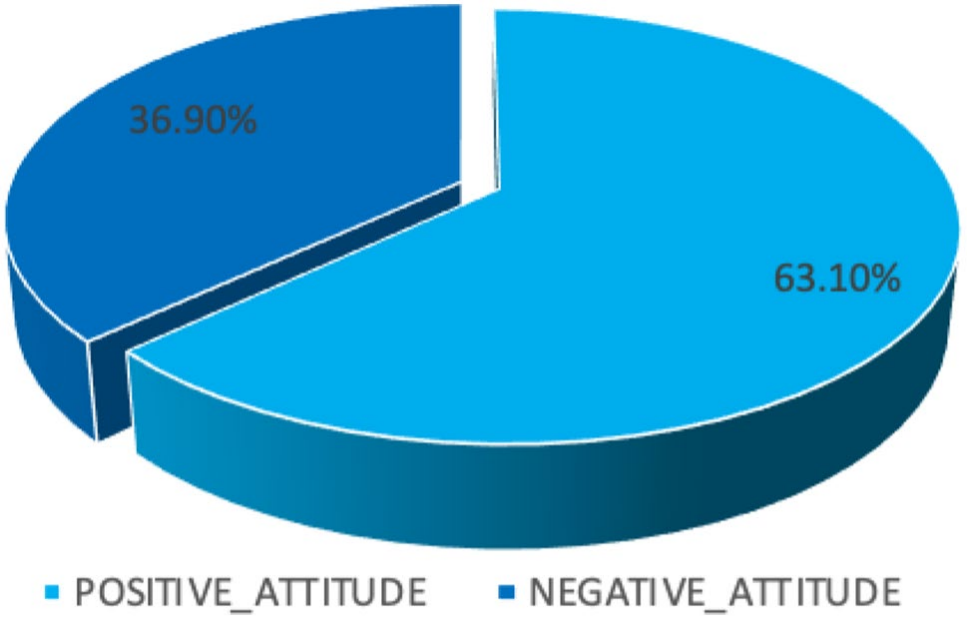
Attitude towards unsafe abortion among study participants

Participants strongly agreed on the following items; unsafe abortion is an unhealthy procedure for a woman 101 (49%), and unsafe abortion can cause complications to a woman 91 (44.2%). The remaining three items indicate that the majority of participants agreed; that unsafe abortion can cause death to a woman 66 (32%), unsafe abortion can lead to sepsis to a woman 68 (33%), and unsafe abortion is a bad thing 75 (36.4%). Refer to Table 5. A total score was computed whereas a score above 10 was a positive attitude toward unsafe abortion while a score below 10 was regarded as a negative attitude toward unsafe abortion. The majority of participants, 130 (63.1%) had positive attitudes toward unsafe abortion compared to those with negative attitudes 76 (36.9%). Refer to Fig. 2.

**Table 5:** Attitude on unsafe abortion among study participants

Variables	Frequency (*n*)	Percentage (%)
**Unsafe abortion is an unhealthy procedure for a woman**		
Strongly disagree	19	9.2
Disagree	10	4.8
Neutral	20	9.7
Agree	56	27.2
Strongly agree	101	49
**Unsafe abortion can cause complications for a woman**		
Strongly disagree	7	3.4
Disagree	22	10.7
Neutral	20	9.7
Agree	66	32
Strongly agree	91	44.2
**Unsafe abortion can cause death to a woman**		
Strongly disagree	14	6.8
Disagree	29	14.1
Neutral	35	17
Agree	66	32
Strongly agree	62	30.1
**Unsafe abortion can lead to sepsis in a woman**		
Strongly disagree	12	5.8
Disagree	33	16
Neutral	30	14.6
Agree	68	33
Strongly agree	63	30.6
**Unsafe abortion is a bad thing**		
Strongly disagree	16	7.8
Disagree	18	8.7
Neutral	31	15
Agree	75	36.4
Strongly agree	66	32

### The association between sociodemographics and the attitude of participants

None of the sociodemographic characteristics was significantly associated with attitude towards unsafe abortion among participants; age group (χ^2^ *=* 10.216; *P* = 0.069), place of residence (χ^2^ *=* 2.571; *P* = 0.109), marital status (χ^2^ *=* 4.801; *P* = 0.187), Occupation (χ^2^ *=* 2.453; *P* = 0.484), and education level (χ^2^ *=* 2.853; *P* = 0.415). Refer to Table 6.

**Table 6:** The association between sociodemographics and attitude of participants

Variables	Attitude		χ^2^ (*P*-Value)
**Age group**	**Positive n (%)**	**Negative n (%)**	
15–19	9 (6.9)	8 (10.5)	10.216 (0.069)
20–24	24 (18.5)	10 (13.2)	
25–29	36 (27.7)	20 (26.3)	
30–34	26 (20)	26 (34.2)	
35–39	20 (15.4)	10 (13.2)	
40–44	15 (11.5)	2 (2.6)	
**Place of residence**			
Rural	25 (19.2)	22 (28.9)	2.571 (0.109)
Urban	105 (80.8)	54 (71.1)	
**Marital status**			
Single	18 (13.8)	15 (19.7)	4.801 (0.187)
Married	84 (64.6)	49 (64.5)	
Divorced	22 (16.9)	6 (7.9)	
Widow	6 (4.6)	6 (7.9)	
**Occupation**			
Peasant	41 (31.5)	26 (34.2)	2.453 (0.484)
Employed	24 (18.5)	8 (10.5)	
House wife	16 (12.3)	9 (11.8)	
Entrepreneur	49 (37.7)	33 (43.4)	
**Education level**			
Not attended formal education	27 (20.8)	10 (13.2)	2.853 (0.415)
Primary school	53 (40.8)	39 (51.3)	
Secondary school	35 (26.9)	19 (25)	
College/university	15 (11.5)	8 (10.5)	

### The association attitude and practice of unsafe abortion

The results show that the attitude towards unsafe abortion was not significantly associated with the practice χ^2^ = 1.066^;^*P* = 0.302.

### Subjective norms towards unsafe abortion

**Fig. 3:**
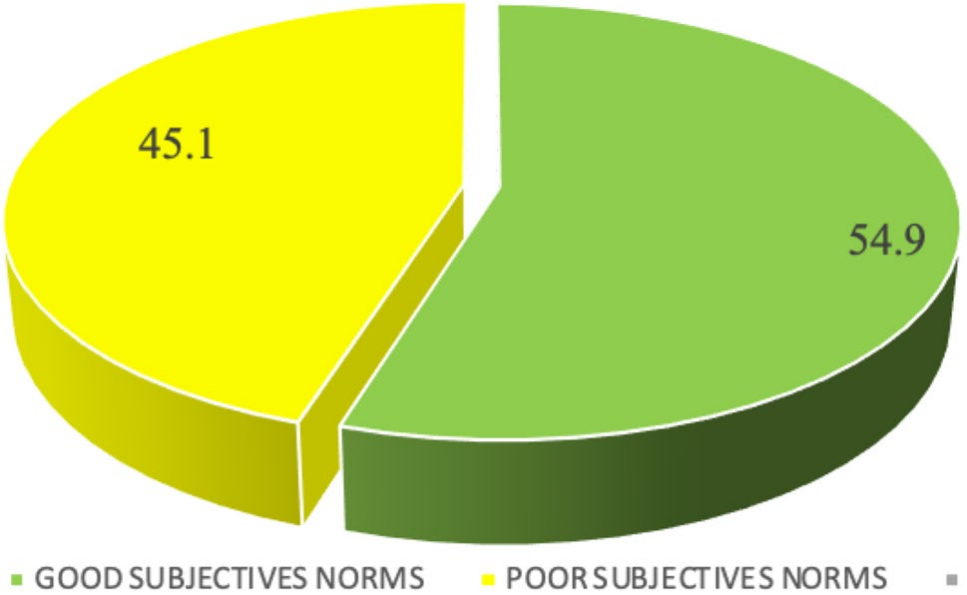
Subjective norms towards unsafe abortion among study participants

Participants answered correctly on the following items: in your family and society is unsafe abortion viewed as a sin 123 (59.7%), When someone is known she had an abortion she is viewed as a killer 129 (62.6%), and when someone is known she had an abortion she is viewed as bad person 120 (58.3%). However, in two items, participants were incorrectly; when someone is known she had an abortion should be stigmatized 118 (57.3%) and do social norms a source of information about unsafe abortion 113 (54.9%). Refer to Table 7. A total score was computed whereas a score above 2 was regarded as good subjective norms toward unsafe abortion while a score below 2 was regarded as poor subjective norms. Through summation of scores, 113 (54.9%) had good subjective norms compared to 93 (45%) who had poor subjective norms. Refer to Fig. 3.

**Table 7:** Subjective norms on unsafe abortion among study participants

Variable	Frequency (*n*)	Percentage (%)
In Your family and society view unsafe abortion viewed as a sin?		
Yes	123	59.7
No	83	40.3
When someone knows she had an abortion should be stigmatized?		
Yes	88	42.7
No	118	57.3
When someone is known she had an abortion she is viewed as a killer.		
Yes	129	62.6
No	77	37.4
When someone is known she had an abortion she is viewed as a bad person		
Yes	120	58.3
No	86	41.7
Social norms is a source of information on unsafe abortion		
Yes	93	45.1
No	113	54.9

### The association between sociodemographic and subjective norms of participants

There were no sociodemographic characteristics significantly associated with subjective norms towards unsafe abortion; age group (χ^2^ *=* 3.732; *P* = 0.589), place of residence (χ^2^ *=* 3.031; *P* = 0.082), marital status (χ^2^ *=* 3.175; *P* = 0.365), occupation (χ^2^ *=* 7.749; *P* = 0.051), and education level (χ^2^ *=* 6.190; *P* = 0.103). Refer to Table 8.

**Table 8:** The association between sociodemographics and subjective norms of participants

Variables	Subjective norms		χ^2^ (*P*-Value)
	Good n (%)	Poor n (%)	
**Age group**			
15–19	11 (9.7)	6 (6.5)	3.732 (0.589)
20–24	19 (16.8)	15 (16.1)	
25–29	33 (29.2)	23 (24.7)	
30–34	28 (24.8)	24 (25.8)	
35–39	16 (14.2)	14 (15.1)	
40–44	6 (5.3)	11 (11.8)	
**Place of residence**			
Rural	31 (27.4)	16 (17.2)	3.031 (0.082)
Urban	82 (72.6)	77 (82.8)	
**Marital status**			
Single	15 (13.3)	18 (19.4)	3.175 (0.365)
Married	72 (63.7)	61 (65.6)	
Divorced	19 (16.8)	9 (9.7)	
Widow	7 (6.2)	5 (5.4)	
**Occupation**			
Peasant	38 (33.6)	29 (31.2)	7.749 (0.051)
Employed	24 (21.2)	8 (8.6)	
House wife	11 (9.7)	14 (15.1)	
Entrepreneur	40 (35.4)	42 (45.2)	
**Education level**			
Not attended formal education	14 (12.4)	23 (24.7)	6.190 (0.103)
Primary school	51 (45.1)	41 (44.1)	
Secondary school	33 (29.2)	21 (22.6)	
College/university	15 (13.3)	8 (8.6)	

### The association of subjective norms and the practice of unsafe abortion

Among participants with good subjective norms, 65 (69.9%) had unsafe abortion. Those who had poor subjective norms, 82 (72.6%) had unsafe abortions. Generally, the results indicate that subjective norms towards unsafe abortion were not significantly associated with the practice (χ^2^ = 0.178^;^*P* = 0.673).

### Perceived behavioral control

Participants agreed on items stating that I cannot practice unsafe abortion because it is illegal in Tanzania 70 (34%) while many participants 58 (28.2%) disagreed on the item “I’m confident that I cannot practice unsafe abortion”. Participants were neutral 54 (26.2%) and others agreed 54 (26.2%) when responding to the item “Regardless I get unwanted pregnancy I cannot practice unsafe abortion”. Refer to Table 9. A total score was computed whereas a score above 6 was regarded as positive perceived behavior control toward unsafe abortion and a score below 6 was regarded as negative perceived behavior control toward unsafe abortion. After the summation of all scores, most of the participants 111 (53.9%) had positive perceived behavioral control while 95 (46.1%) had negative perceived behavioral control.

**Table 9:** Perceived behavior control on unsafe abortion among study participants

Variables	Frequency (*n*)	Percentage (%)
I’m confident that I cannot practice unsafe abortion		
Strongly disagree	17	8.3
Disagree	58	28.2
Neutral	54	26.2
Agree	57	27.7
Strongly agree	20	9.7
Regardless I get an unwanted pregnancy I cannot practice unsafe abortion		
Strongly disagree	21	10.2
Disagree	34	16.5
Neutral	54	26.2
Agree	54	26.2
Strongly agree	43	20.9
I cannot practice unsafe abortion because it is illegal in Tanzania		
Strongly disagree	12	5.8
Disagree	31	15
Neutral	45	21.8
Agree	70	34
Strongly agree	48	23.3

### The association between sociodemographic and perceived behavioral control of participants

It was found that none of the sociodemographic characteristics of participants was significantly associated with perceived behavioral control; age group (χ^2^ *=* 5.849; *P* = 0.321), place of residence (χ^2^ *=* 2.075; *P* = 0.15), marital status (χ^2^ *=* 2.595; *P* = 0.458), occupation (χ^2^ *=* 5.384; *P* = 0.146), and education level (χ^2^ *=* 3.905; *P* = 0.272). Refer to Table 10.

**Table 10:** The association between sociodemographic and perceived behavioral control of participants

Variables	Perceived behavioral control		χ^2^ (*P*-Value)
	Positive n (%)	Negative n (%)	
**Age group**			
15–19	6 (5.4)	11 (11.6)	5.849 (0.321)
20–24	17 (15.3)	17 (17.9)	
25–29	28 (25.2)	28 (29.5)	
30–34	34 (30.6)	18 (18.9)	
35–39	16 (14.4)	14 (14.7)	
40–44	10 (9)	7 (7.4)	
**Place of residence**			
Rural	21 (18.9)	26 (27.4)	2.075 (0.15)
Urban	90 (81.1)	69 (72.6)	
**Marital status**			
Single	16 (14.4)	17 (17.9)	2.595 (0.458)
Married	77 (69.4)	56 (58.9)	
Divorced	13 (11.7)	15 (15.8)	
Widow	5 (4.5)	7 (7.4)	
**Occupation**			
Peasant	42 (37.8)	25 (26.3)	5.384 (0.146)
Employed	14 (12.6)	18 (18.9)	
House wife	10 (9)	15 (15.8)	
Entrepreneur	45 (40.5)	37 (38.9)	
**Education level**			
Not attended formal education	20 (18)	17 (17.9)	
Primary school	44 (39.6)	48 (50.5)	3.905 (0.272)
Secondary school	31 (27.9)	23 (24.2)	
College/university	16 (14.4)	7 (7.4)	

### The association of perceived behavioral control and practice of unsafe abortion

The results show that perceived behavioral control towards unsafe abortion was not significantly associated with the practice χ^2^ = 0.466; *P* = 0.495).

### The association of sociodemographic characteristics and practice of abortion

The result indicates that only two sociodemographic characteristics (Marital status and educational level) were significantly associated with the safe abortion. In such a way respondents who were divorced had high good practice 16 (57.1%) compared to other participants who belonged to other marital statuses (χ^2^ = 13.515; *P* = 0.004) and participants who had secondary educational level had safe abortion 73 (79.3%) than other participants with different educational levels (χ^2^ = 10.146; *P* = 0.017). Other sociodemographic characteristics were significantly association with safe abortion; age (χ^2^ = 8.751*P* = 0.119), place of residence (χ^2^ = 0.029; *P* = 0.866), and occupation (χ^2^ = 4.663; *P* = 0.198). Refer to Table 11.

**Table 11:** The association of sociodemographic characteristics and practice of unsafe abortion

Demographic characteristics	The practice of unsafe abortion		χ^2^ (*P*-Value)
	Good practice *n* (%)	poor practice *n* (%)	
**Age group**			
15–19	1(5.9)	16(94.1)	8.751(0.119)
20–24	10(29.4)	24(70.6)	
25–29	12(23.1)	40(76.9)	
30–34	12(23.1)	40(76.9)	
35–39	10(33.3)	20(66.7)	
40–44	4(23.5)	13(76.5)	
**Place of residence**			
Rural	13(27.7)	34(72.3)	0.029 (0.866)
Urban	46(28.9)	113(71.1)	
**Marital status**			
Single	72 (1.2)	26(78.8)	13.515 (0.004)
Married	32 (24.1)	101(75.9)	
Divorced	16 (57.1)	12(42.9)	
Widow	43 (3.3)	8(66.7)	
**Occupation**			
Peasant	16(23.9)	51(76.1)	4.663 (0.198)
Employed	12(37.5)	20(62.5)	
House wife	4(16)	21(84)	
Entrepreneur	2732.9)	55(67.1)	
**Educational level**			
Not attended formal	8(21.6)	29(78.4)	10.145 (0.017)
Primary school	19(20.7)	73(79.3)	
Secondary school	23(42.6)	31(57.4)	
University/college level	9(39.1)	14(60.9)	

### Interaction of knowledge, attitude, and perceived behavioral control

Knowledge level of unsafe abortion was not significantly associated with attitude (χ2 = 3.657; *P* = 0.056). Refer to Supplementary Table (1) In the same way, there was no significant association between knowledge level and perceived behavioral control (χ2 = 3.537; *P* = 0.06). Refer to Supplementary Table (2) Meanwhile, there was a significant association between attitude and perceived behavioral control in such a way that 61 (55%) participants who had a positive attitude towards unsafe abortion had positive perceived behavioral control (χ2 = 3.657; *P* = 0.056). Refer to supplement Table 3.

## Discussion

### Knowledge of unsafe abortion among the participants

The current study found that most of the participants 129 (62.6%) had adequate knowledge of unsafe abortion. This indicates the effectiveness of the ongoing national approaches for promoting public understanding of unsafe abortion. Further, it is because the majority were aged 30–34 and probably had been exposed to previous health education from antenatal care visits. The adequate knowledge of unsafe abortion (91%) is also reported in the previous study [30].

### Attitude on unsafe abortion among participants

In this study, two-thirds of the participants 130 (63.1%) had positive attitudes. health beliefs (perceived susceptibility, perceived severity, perceived benefits) might be a reason for a positive attitude. For instance, when a woman feels susceptible to sepsis after an unsafe abortion might help her to avoid practicing unsafe abortion. Same way, the woman will avoid unsafe abortion if believes that she may end up with complications and unfertile. The previous study supports that health beliefs influence attitude [31]. Moreover, another study pinpoints that health beliefs and attitudes have a significant association with lifestyle change and risks in primary care [32].

### Subjective norms on unsafe abortion among participants

From the results of our study, most participants had good subjective norms toward unsafe abortion 113 (54.9%) because the participants encounter pressure from a society that considers unsafe abortion sin, the doer of unsafe abortion as a killer, and anyone involved in unsafe abortion needs to be stigmatized. The finding is aligned with previous findings by Connell [33].

### Perceived behavior control among participants

From the results of our study, the majority 111 (53.9%) had positive perceived behavior control, which has been influenced by the positive attitude participants possessed. It is surprising to find out that the current study finding is contrary to the theory of planned behaviour since the theory is silent on whether attitude influences perceived behavioral control.

### The practice of unsafe abortion among participants

Most participants 147 (71.4%) were found to practice unsafe abortion due to marital status and educational level. Since most of the participants in this study were single women and abortion in Tanzania is illegal, if a single woman becomes pregnant while still having a plan to develop a career, unsafe abortion is the alternative. Meanwhile, if a single woman becomes pregnant but the pregnancy is rejected by a partner, this triggers the woman to perform an unsafe abortion. Regarding education level, participants with lower educational levels or those who have never attended the school practice unsafe abortion. The school is the place with health clubs, teaching, and guiding students in all health-related matters with unsafe abortion inclusively. The more a woman advances her education level, the more she is exposed to teachings on unsafe abortion. Out of health clubs, the curriculum may contain courses with the content of unsafe abortion. The finding is consistent with the previous study reported that unsafe abortion is higher among less educated women [34].

### Study limitation

The study was carried out in a single region of Tanzania and at a single healthcare facility which may limit the generalization of the current findings. It seems that most of the important factors for unsafe abortion were left out as none of the independent variables in the current study were significantly associated with the practice of unsafe abortion.

## Conclusion

Most of the respondents had high knowledge, attitudes, subjective norms, and perceived behavior control on unsafe abortion. This is an indicator that the implemented initiatives are effective. Maintaining the ongoing effort and improving strategies are promising to mitigate the burden of unsafe abortion. Future research needs to find out hidden factors associated with attitude and how health beliefs might influence someone’s attitude towards unsafe abortion. Even though the theory does not support the attitude to influence perceived behavioral control, extensive studies need to be conducted to confirm the association between these two variables.

### Electronic supplementary material

Below is the link to the electronic supplementary material.

Supplementary Material 1

## Data Availability

The data set and other supplementary documents are available upon request. Point of contact, Dr. Joanes Faustine Mboineki, Email: 624639045@qq.com, Mobile number: +255756310634.
