# Supplementary material for: Knowledge level and constructs of the theory of planned behaviour (TPB) to the practice of unsafe abortion among postnatal mothers attending Mkonze health center, Dodoma Region, Tanzania
Source: BMC Public Health. 2024 May 28;24:1427. doi: 10.1186/s12889-024-18921-z (PMC11134745; doi:10.1186/s12889-024-18921-z)
Supplement: Supplementary file 1 — Supplementary Material 1 [file 12889_2024_18921_MOESM1_ESM.doc]

# Supplementary Table 1: Interaction of knowledge and attitude

| **Variables** | **Attitude** | | **χ^2^ (*P*-Value)** |
| --- | --- | --- | --- |
| **Knowledge** | **Positive n (%)** | **Negative n (%)** |  |
| Adequate knowledge | 75 (57.7) | 54 (71.1) | 3.657 (0.056) |
| Inadequate knowledge | 55 (42.3) | 22 (28.9) |  |
|  |  |  |  |

# Supplementary Table 2: Interaction of knowledge and perceived behavioral control

| **Variables** | **Perceived behavioral control** | | **χ^2^ (*P*-Value)** |
| --- | --- | --- | --- |
| **Knowledge level** | **Positive n (%)** | **Negative n (%)** |  |
| Adequate knowledge | 63 (56.8) | 66 (69.5) | 3.537 (0.06) |
| Inadequate knowledge | 48 (43.2) | 29 (30.5) |  |
|  |  |  |  |

# Supplementary Table 3: Interaction of attitude and perceived behavioral control

| **Variables** | **Perceived behavioral control** | | **χ^2^ (*P*-Value)** |
| --- | --- | --- | --- |
| **Attitude** | **Positive n (%)** | **Negative n (%)** |  |
| Positive attitude | 61 (55) | 69 (72.6) | 6.87 (0.009) |
| Negative attitude | 50 (45) | 26 (27.4) |  |
